# AHRQ’s digital healthcare research program: 20 years of advancing innovation and discovery

**DOI:** 10.1093/jamia/ocae251

**Published:** 2024-09-30

**Authors:** R Burciaga Valdez, Christine Dymek, Kevin Chaney, Edwin A Lomotan

**Affiliations:** Agency for Healthcare Research and Quality, Rockville, MD 20857, United States; Agency for Healthcare Research and Quality, Rockville, MD 20857, United States; Agency for Healthcare Research and Quality, Rockville, MD 20857, United States; Agency for Healthcare Research and Quality, Rockville, MD 20857, United States

**Keywords:** digital healthcare research, health information technology, patient safety, artificial intelligence, telehealthcare

## Abstract

**Objectives:**

To reflect on the achievements of the Agency for Healthcare Research and Quality’s (AHRQ) Digital Healthcare Research Program over the past 20 years, evaluate its impact on US healthcare quality and safety, and outline current and future priorities for digital healthcare research and innovation.

**Process:**

The article reviews key milestones in AHRQ’s digital healthcare initiatives, including its founding and its advances in telehealthcare and clinical decision support. It highlights AHRQ’s contributions to advancing technology integration in healthcare, promoting patient safety, and addressing equity gaps. The article also examines the evolving role of artificial intelligence (AI) in healthcare delivery.

**Conclusions:**

AHRQ’s Digital Healthcare Research Program has significantly contributed to improving healthcare quality. As digital technologies evolve, particularly with AI, the program remains focused on enhancing safety, equity, and efficiency in healthcare. Continued research and investment will be essential to maintaining progress and addressing new challenges.

## Introduction

The US healthcare system’s quality and safety deficiencies are well documented.^1–4^ These deficiencies include basic failures to provide care that is uniformly safe, timely, effective, efficient, equitable, and patient-centered.^1^ To address these quality and safety gaps—gaps so large that they were deemed a “chasm”^2^—healthcare provider organizations, clinicians, and policymakers have increasingly turned to digital healthcare technologies. Electronic health record (EHR) systems, telehealthcare services, clinical decision support (CDS) systems, and other technology-driven innovations have demonstrated significant potential to improve healthcare quality and safety in the United States.^5^

These technologies are not cure-alls. But some of them are already transforming care that gets closer to the ideal of high quality: delivered in a patient-centered, timely, safe, effective, and efficient manner that is equitable.^6^

The Agency for Healthcare Research and Quality (AHRQ) has been at the forefront of this transformation. AHRQ’s unique federal mission is to improve the quality of healthcare available to all in our nation.^7^ AHRQ strives to turn scientific evidence into “actionable knowledge” and develops the tools and data needed to improve healthcare system performance and help patients, clinicians, and policymakers make informed decisions. AHRQ’s health services research portfolio includes a series of digital healthcare interventions that have become foundational to the US healthcare quality and safety enterprise.

This year, AHRQ’s Digital Healthcare Research Program marks its 20th anniversary of improving healthcare quality through research and support of the implementation of innovative technology-based tools and resources.^8^ In doing so, we reflect on what we have accomplished and learned, highlight some of our most important current work, and propose a digital healthcare-driven vision for the future. (A timeline of key achievements of this program can be found at https://digital.ahrq.gov/dhr-20.)

We note that AHRQ’s digital healthcare research accomplishments were achieved with important work supported by other federal and state agencies, private foundations, and healthcare membership associations. We gratefully acknowledge these partners, especially our numerous sister agencies within the Department of Health and Human Services and other federal agencies that have tirelessly worked to advance this important research. Nevertheless, many digital healthcare interventions that are commonplace today originate from AHRQ initiatives. AHRQ-funded research has helped create the foundation that comprises the US digital healthcare ecosystem.^9–11^

## The early years: advances in telehealthcare, health information exchange, and patient safety

AHRQ’s history in supporting research on digital healthcare, alternatively known as medical informatics or health information technology (IT), predates the modern agency itself. It begins with AHRQ’s predecessor entities, the National Center for Health Services Research and Development (beginning in 1968), the National Center for Health Services Research and Health Care Technology Assessment (from 1985 through 1988), and the Agency for Health Care Policy and Research (from 1989 to 1999).^12–14^ The focus of these entities’ early research funding in the field was on acquiring and communicating patient data to improve quality and make care more efficient.^14^

Congress reauthorized and renamed AHRQ in 1999.^15^ In doing so, it directed AHRQ to evaluate technologies to promote quality and safety improvement.^15^^,^^16^ AHRQ’s first significant foray into digital healthcare research in the 21st century followed quickly: the Clinical Informatics to Support Patient Safety (“CLIPS”) research initiative in 2001.^16^ This initiative drove research investment in the patient safety and health IT research communities and resulted in competitive proposals from various organizations. Projects funded through this grant initiative included those on improving primary care patient safety with decision support systems, mining complex clinical data for patient safety research, and using technology to reduce errors in attention-deficit/hyperactivity disorder care.

By 2004, recognition was growing that health IT required a tighter focus on healthcare quality and patient safety. So, the agency established the Division of Health Information Technology, the precursor to today’s Digital Healthcare Research Program.

The new division’s first act was to embark on a series of grants called Transforming Healthcare Quality through Health Information Technology, or THQIT.^17^ Under the THQIT initiative, 118 grantees planned, implemented, and studied the value of digital healthcare broadly. THQIT-funded projects spanned a gamut of digital healthcare innovations, including electronic prescribing and the first projects using web-based systems to improve care.

THQIT’s crown jewel was its achievement in telehealth consultation, a precursor to today’s telehealthcare. AHRQ was an early proponent of this concept and supported it with a 2004 grant that became the foundation of Project ECHO (Extension for Community Healthcare Outcomes),^18^ an early telehealth consultation model. This research project demonstrated that the care available in cities with specialty medical practices could be achieved at the same level in rural areas by bringing rural doctors and University of New Mexico disease specialists together online to co-manage chronic disease patients. In doing so, Project ECHO demonstrated that telehealth consultation could succeed broadly. ECHO remains the model on which a significant amount of telehealthcare infrastructure is based.^19^ It has continued to influence care—notably as a central aspect of the federal government’s response to the COVID-19 pandemic.^20^

During these early years, AHRQ pursued technology-focused work in several other areas. These included:

- **State and Regional Health Information Exchange Projects**. AHRQ funded 6 states to identify and support data sharing and interoperability activities to improve healthcare for patients and populations at a state or regional level. They found that health information exchanges (HIEs) were critical in facilitating seamless health data exchange across disparate healthcare systems.^21^ These initial efforts laid the groundwork for today’s Qualified Health Information Networks (QHINs), which are networks of organizations that share health data through the Assistant Secretary for Technology Policy (ASTP)/Office of the National Coordinator for Health Information Technology (ONC)’s Trusted Exchange Framework and Common Agreement.^22^ QHINs are an evolution of HIEs, operating on a larger, often national scale and providing more comprehensive data sharing capabilities.
- **Health Information Technology in Rural Hospitals.** This project met the unique needs of rural hospitals with planning health IT enhancements to address patient safety and care quality issues. It developed toolkits to guide health IT investment and implementation decisions that fit the rural healthcare environment.^23^^,^^24^
- **CDS and Computerized Provider Order Entry (CPOE).** AHRQ pursued more than 20 projects dedicated to demonstrating the value of health IT,^25^ with a particular focus on CDS^26^ and CPOE^27^ technologies that have become cornerstones of modern healthcare. AHRQ’s predecessor entities were funding CDS research as early as the 1980s.^14^ Later, AHRQ-funded research led to a widely quoted article in this journal, the “Ten Commandments for Clinical Decision Support.”^28^ This accelerated in 2004 with a project to design and implement “smart forms” and quality dashboard tools and to assess their impact on guideline adherence.^29^ Also in 2004, a project to implement CPOE in intensive care units^27^ revealed human factors challenges in employing order entry with EHR systems. These accomplishments, together with the use of emerging technologies (eg, new channels and formats) in delivering key information to clinicians at the point of care, laid the groundwork for tremendous advancement in EHR functionality and standards-based CDS.

## Continuing to grow: decision support and workflow integration

By 2008, AHRQ’s Division of Health Information Technology had become an established leader in the field of clinical informatics research. It grew by funding 2 important CDS demonstration projects: the Clinical Decision Support Consortium,^30^ which included clinical institutions and technology vendors to define best practices for CDS in ambulatory care; and the GLIDES (GuideLines Into Decision Support) project,^31^ which implemented CDS tools for evidence-based guideline recommendations for pediatric obesity and asthma. These demonstrations became the foundation for many of the scalable, standards-based CDS applications that are more common today.

During this time, AHRQ focused on building a greater understanding of health IT’s impact on end users, while also incorporating cognitive and human factors engineering approaches to inform the future design and integration of health IT.^32^

- **Incorporating Health Information Technology into Workflow Redesign.** AHRQ funded a contract that studied the existing research related to the impacts of health IT on workflow in outpatient settings and how health IT can be used to assess workflow in these settings. The information led to the development of a toolkit^33^ that included instruments, methods, and strategies to understand and assess workflow, inform workflow issues, and recognize how workflow can be impacted by implementation and use of health IT.
- **Advancing Health Information Technology Design and Integration.** AHRQ funded 28 grants aimed at growing the knowledge base to better understand the information needs of patients^34^ and clinicians^35^ in the context of health IT, and 3 contracts^36–38^ focused on the impact of health IT on workflow and practice redesign.

The collective work contributed to growing the field’s knowledge and understanding to improve the design, integration, assessment, and refinement of health IT.

In 2014, the Division and the ONC, with support from the Robert Wood Johnson Foundation (RWJF), published 2 reports by JASON, an independent group of expert scientists and academicians, that would influence the development of the field: one on health data infrastructure,^10^ the other on individual health data.^11^ The former report’s recommendations informed ONC’s Shared Nationwide Interoperability Roadmap,^39^ while the latter called for greater integration of other health data types. Both reports remain influential for the data infrastructure necessary to enable a learning health system.

By 2020, AHRQ’s Division of Health Information Technology had matured into an essential part of the agency’s research program. Reflecting this newfound centrality to the healthcare quality enterprise and growth within the field itself, the division renamed itself the Digital Healthcare Research Program. This signified a strategic realignment with the evolving digital healthcare landscape, with the term “digital healthcare” applying to activities involving the transfer of information between patient and provider throughout the entire patient journey and the intelligent use of all related data.^40^

## The digital healthcare research program today: leading innovation

Today, the Digital Healthcare Research Program is a beacon of innovation, driving significant advancements in digital healthcare that respond to the needs of patients and healthcare providers. In the 20 years since it was established, the program has invested $711 million in 740 grants and contracts.^41^ The program’s publicly available, searchable database, which can be found at https://digital.ahrq.gov/ahrq-funded-projects/search, captures the totality of these projects, which span nearly every aspect and setting of care.

The program has had an outsized impact on the field’s top researchers, with more than half of the winners of the American College of Medical Informatics’ prestigious Morris F. Collen Award of Excellence having received AHRQ funding.^42^ The program’s initiatives are shaping the future of healthcare by using technology to enhance quality and patient safety while advancing health equity.

The Digital Healthcare Research Program’s work is organized around 5 priorities. They are:

1. **Digital healthcare equity.** The Digital Healthcare Research Program is dedicated to advancing healthcare quality through an equity lens. Recognizing the crucial role of social and digital determinants of health, the program supports research on integrating these data into clinical settings effectively. This includes research comparing predictive modeling to questionnaire-based screening for assessing social needs. Additionally, in April 2024, AHRQ published a Digital Healthcare Equity Framework^43^ and Guide^44^ to ensure equity is prioritized across all phases of the digital healthcare lifecycle.
2. **Virtual healthcare.** The COVID-19 pandemic drove a surge in virtual healthcare, particularly telehealthcare. This led to pandemic-specific AHRQ research grants^45^ with a focus on telehealthcare. AHRQ’s work in virtual healthcare remains vibrant; examples include a current project focused on remote lung cancer surveillance^46^ and exploring improved and novel healthcare delivery approaches.^47^^,^^48^ This remains a significant growth area for the field for various reasons, including improving access.
3. **Patient-generated health data (PGHD) and patient-reported outcomes (PROs).** Remote patient monitoring that utilizes electronically gathered PGHD, including PROs, is an important complement to clinical care, especially telehealthcare. AHRQ is interested in research exploring the efficacy and safety of digital healthcare innovations that utilize virtually gathered PGHD. AHRQ developed a PGHD guide^49^ to support ambulatory practices in collecting and responsibly using these data and guidelines to facilitate meaningful and effective PRO use in dynamic, real-world clinical settings.^50^
4. **CDS.** Technologies integrating CDS into EHR systems and encouraging the adoption of evidence-based guidelines remain foundational to the digital healthcare research enterprise. AHRQ’s ongoing CDS work includes research to enhance CDS effectiveness and integrate patient-centered outcomes research findings into practice through interoperable CDS. Meanwhile, AHRQ-funded researchers have defined a vision for CDS over the next 25 years,^51^ emphasizing the need for systems that react to the inputs of healthcare professionals and are also proactive in anticipating patients’ needs. Finally, AHRQ’s CDS Innovation Collaborative^52^ works with stakeholders to advance patient-centered CDS through measurement frameworks, prototype dashboards, and pilot projects.
5. **Safety.** Research is crucial in understanding how digital healthcare tools can enhance patient safety and be safely implemented. AHRQ-funded research explores how patient photos in the EHR can reduce the risk of wrong-patient errors,^53^ develops new medication safety measures,^54^ and identifies safety gaps to inform guidelines for system implementation.^55^ This practical evidence is vital for health systems to make informed decisions and follow best practices for implementing and using digital healthcare resources to promote safety.

Each of these areas of study has some overlap with the others, and yet they are distinct. These priorities reflect AHRQ’s commitment to advancing health services research to make healthcare more accessible, equitable, affordable, and safer.^56^

Each year, the program publishes a Year in Review summarizing its work and showcasing the impact of its research projects.^57^ The Year in Review reports identify themes from emerging research, spotlight individual research projects and principal investigators, and describe the impact of the resources and research results that are disseminated throughout the year. The most current Year in Review is available at https://digital.ahrq.gov/program-overview/research-reports.

## A look ahead: the digital healthcare research program vision coming into focus

One must consider artificial intelligence (AI) before one can think about the future of healthcare. While AI adoption in healthcare may be lagging behind other industries,^58^ it is nevertheless already revolutionizing the field. For instance, the 2023 publication of a paper written mainly by ChatGPT in a medical journal^59^ demonstrates the power of commonly available AI technologies to produce evidence at a pace previously considered unthinkable, while the founding of a new medical journal explicitly devoted to the use of AI in healthcare^60^ demonstrates the seriousness with which the academic community is approaching the matter. The application of AI in clinical decision-making heralds a new era in which algorithms can analyze vast amounts of data to provide clinicians with actionable insights, enhancing diagnostic accuracy and optimizing treatment pathways.

The prospect of unchecked AI in healthcare may make some uneasy. Continued research and attention are required to mitigate the technology’s potential negative consequences, such as promoting false information or widening equity gaps.

Fortunately, AHRQ has already considered how AI affects care delivery. AHRQ has investigated AI since its very early development.^61^ In 2017, the Division, in conjunction with ONC and RWJF, published a third study by JASON. In this report, scientists and experts assessed the realistic implications of AI applications for health and healthcare.^62^ This report concluded that broad advances in AI are significant and real—a conclusion that, 7 years later, is no longer controversial. This report also cautioned about the limitations of AI, the need for quality data, and the need to foster capabilities to capture and integrate data on environmental exposures and social behaviors in AI healthcare applications because health outcomes are affected by these factors.

More recently, the agency has funded or is currently funding more than 50 research projects with an AI component.^63^ These comprise approximately 17% of the Digital Healthcare Research Program’s new grants since 2020^64^ and cover many technologies and aspects of care, including CDS,^65^^,^^66^ cancer screening,^67^^,^^68^ medication safety,^69^^,^^70^ and adaptive mobile messaging.^71^ AHRQ-funded projects use AI to improve emergency department care, refine workflow, and reduce clinical burden. In July 2024, AHRQ published a new Notice of Funding Opportunity (NOFO) to accelerate this research.^72^ This NOFO seeks to determine whether and how certain breakthrough uses of AI systems can affect patient safety, and how AI systems can be safely implemented and used.

AI has the potential to improve the safety, effectiveness, efficiency, accessibility, and affordability of healthcare. However, as with most technologies, we must be cautious. We must identify and mitigate risks for patient harm and user burden. In particular, AI will likely profoundly impact telehealthcare, spurring it to include more remote monitoring, disease surveillance, and personalized care in rural and underserved communities.^73^ It is necessary to ensure that as AI is developed and adopted, we need to keep equity in mind and ensure that AI does not contribute to or create new digital divides.

Improving the quality of American healthcare remains AHRQ’s North Star. The Digital Healthcare Research Program’s work is fully aligned and intertwined with this. The program remains committed to advancing safety, efficiency, and equity by supporting research and implementation projects that develop new technologies and apply existing ones in real-world care settings. The lessons we learn from this are then translated into usable tools that bring digital healthcare interventions to patients for their direct benefit.

At 20, AHRQ’s Digital Healthcare Research Program is just starting. Our work will never be complete because healthcare quality improvement is the veritable “never-ending journey”^74^; however, every day, we get a little closer to achieving the 6 domains of quality that the Institute of Medicine spelled out decades ago.^2^ We hope you will join us on the journey. Please visit https://digital.ahrq.gov to learn more about the AHRQ Digital Healthcare Research Program and, for potential research support, www.ahrq.gov/funding/fund-opps/index.html to learn about Notices of Funding Opportunities.

## Data Availability

No new data were generated in the writing of this article. All underlying information can be found at https://ahrq.gov and at https://digital.ahrq.gov.
